# Neutrophil-to-Lymphocyte Ratio Predicts Contrast-Induced Acute Kidney Injury in Patients with ST-Elevation Myocardial Infarction Treated with Primary Percutaneous Coronary Intervention

**Published:** 2019-04

**Authors:** Veysel Ozan Tanık, Tufan Çınar, Yalçın Velibey, Ahmet Öz, Koray Kalenderoğlu, Ayça Gümüşdağ, Emre Aruğaslan, Muhammed Keskin, Mehmet Eren

**Affiliations:** 1 *Department of Cardiology, Ankara Dışkapı Yıldırım Beyazıt Training and Research Hospital, Ankara, Turkey.*; 2 *Department of Cardiology, Sultan Abdülhamid Han Training and Research Hospital, Health Sciences University, Istanbul, Turkey.*; 3 *Department of Cardiology, Siyami Ersek Training and Research Hospital, Health Sciences University, Istanbul, Turkey.*; 4 *Department of Cardiology, Sivas Numune Hospital, Sivas, Turkey.*

**Keywords:** *Acute kidney injury*, *Myocardial infarction*, *Percutaneous coronary intervention*, *Neutrophils*, *Lymphocyte count*

## Abstract

**Background**: Development of contrast-induced acute kidney injury (CI-AKI) in patients with ST-elevation myocardial infarction (STEMI) treated via primary percutaneous coronary intervention (PCI) is a major cause of morbidity and mortality worldwide. The neutrophil-to-lymphocyte ratio (NLR), which is a marker of inflammation, has been demonstrated to be associated with the development of major adverse cardiovascular outcomes in many studies. From this point of view, in this study, we aimed to evaluate the predictive value of the NLR as regards the occurrence of CI-AKI in patients with STEMI undergoing primary PCI.

**Methods**: This study was conducted at Dr. Siyami Ersek Training and Research Hospital from May 2008 to June 2016. A total of 2000 patients with STEMI treated via primary PCI were enrolled in the study. The NLR was calculated as the ratio of the number of neutrophils to the number of lymphocytes. All venous blood samples were obtained within 8 hours after admission. CI-AKI was the primary end point of the study. Then, the relationship between CI-AKI and the NLR was assessed.

**Results**: CI-AKI was detected in 148 (7.4%) patients. The patients who developed CI-AKI had a significantly higher NLR than those who did not (7.08±4.43 vs. 6.18±3.98; P=0.011). In the multivariate logistic regression analyses, the NLR remained a significant independent predictor of CI-AKI (OR: 1.78, 95% CI: 1.21–2.61, and P=0.003).

**Conclusion**: The NLR may be a significant independent predictor of CI-AKI in patients with STEMI treated via primary PCI and higher NLR values could be independently associated with a greater risk for CI-AKI.

## Introduction

Contrast-induced acute kidney injury (CI-AKI) is a well-known and important complication in patients who undergo primary percutaneous coronary intervention (PCI) for ST-segment elevation myocardial infarction (STEMI).^1^^-^^3^ The development of CI-AKI after primary PCI is associated with recurrent revascularization procedures, increased mortality and morbidity, and prolonged hospitalization.^4^^, ^^5^ Depending on the definition criteria applied, the incidence of CI-AKI has varied from roughly 6.4% to as high as 27.7%.^1^ The possible mechanisms that are connected with the development of CI-AKI after primary PCI include direct tubular epithelial cell injury due to contrast media, intra-renal vasoconstriction, oxidative stress, medullary hypoxia, endothelial dysfunction, inflammation, a reduced renal blood flow, and the generation of reactive oxygen species.^5^^-^^11^

The neutrophil-to-lymphocyte ratio (NLR), which is a marker of inflammation, has been associated with the development of major adverse cardiovascular outcomes in many studies.^12^^, ^^13^ An elevated NLR is an important independent predictor of short- and long-term survival and the no-reflow development in patients with STEMI.^14^ In light of these data, we may claim that the development of CI-AKI in patients with STEMI treated via primary PCI is an inflammatory process. Thus, in this study, we aimed to investigate the relationship between the development of CI-AKI and the NLR on admission in patients admitted with STEMI undergoing primary PCI.

## Methods

A total of 2400 patients who were diagnosed with STEMI in Dr. Siyami Ersek Training and Research Hospital between May 2008 and June 2016 and underwent primary PCI were retrospectively analyzed. The exclusion criteria were comprised of known hypersensitivity to contrast media and statins, pregnancy or breastfeeding, cardiogenic shock, cardiopulmonary resuscitation due to cardiopulmonary arrest, end-stage renal failure (estimated glomerular filtration rate [eGFR] <15 mL/min/l.73 m^2^ or dialysis treatment), autoimmune diseases, active cancer, severe liver diseases, use of nephrotoxic drugs (e.g., nonsteroidal anti-inflammatory drugs) in the preceding 7 days, clinically evident acute or chronic inflammation, and mechanical complications within 72 hours. After the evaluation regarding the exclusion criteria, 2000 patients with STEMI were enrolled in the study.

STEMI was defined as follows: I) at least 2 contiguous leads with ST-segment elevations greater than 2.5 mm in men below 40 years of age, greater than 2 mm in men over 40 years of age, or greater than 1.5 mm in women in the leads V_2_ to V_3_ and/or greater than 1 mm in the other leads (in the absence of left ventricular hypertrophy or left bundle branch block); II) a prolonged (>30 min) typical chest pain at rest; and III) increased serum biomarkers of myocardial damage.^15^ For the patients diagnosed before 2012, the following cutoff points were used to define a persisting ST elevation, in compliance with the previous universal definition of MI: greater than 0.1 mV in all leads other than leads V_2_ to V_3_, where the following cutoff points apply: greater than 0.2 mV in men, greater than 0.15 mV in women, or a new-onset left bundle branch block.^16^ The study was approved by the local ethics committee, and written informed consents were obtained from all the patients.

Transthoracic echocardiography was performed in all the patients within 48 hours after admission by using GE Vivid 5 and 7 systems (GE Vivid 5 and 7; GE Healthcare, Piscataway, New Jersey) echocardiography machines. The left ventricular ejection fraction (LVEF) of each patient was calculated by using the biplane Simpson method. A 2.5- to 3.5-MHz phased-array transducer was used, and systolic dysfunction was defined as an LVEF below 40%.

Venous blood samples were obtained from all the patients within 8 hours after admission. White blood cell, neutrophil, lymphocyte, monocyte, and platelet counts and hemoglobin levels were measured as part of the automated complete blood count using a Cell-Dyn 37000 Hematology Analyzer (Abbott Diagnostic Division, Wiesbaden, Germany). The NLR was calculated as the ratio of the number of neutrophils to the number of lymphocytes. Blood urea nitrogen and serum creatinine levels were measured as part of biochemical parameters using an Architect Plusci 4100 (Abbott Laboratories, Abbott Park, Illinois). The eGFR was calculated by using the Chronic Kidney Disease Epidemiology Collaboration’s creatinine equation.^17^

Coronary angiography was performed via the femoral artery in all the patients within 90 minutes after admission. All the patients received 300 mg of acetylsalicylic acid and 300 to 600 mg of an oral loading dose of clopidogrel on admission. Clopidogrel (75 mg) was continued for at least 12 months after PCI, and acetylsalicylic acid (100 mg) was indefinitely prescribed. Standard intravenous bolus unfractionated heparin (70–100 U/kg) and additional doses as needed were given in order to achieve an activating clotting time of greater than 250 seconds before the coronary intervention. The addition of a GpIIb-IIIa inhibitor (tirofiban, Aggrastat [12.5 mg/50 mL]; DSM Pharmaceuticals, Greenville, North Carolina) was not obligatory and was left to the operator’s judgment per institutional protocol.

CI-AKI was described as a higher than 25% relative increase or a higher than 0.5 mg/dL absolute increase in serum creatinine above baseline within 72 hours after primary PCI.^18^ Chronic renal failure was defined as having an eGFR below 60 mL/min/1.73 m^2^ for over 3 months with or without kidney damage.^19^ Anemia was defined as a baseline hemoglobin value of below 13 g/dL for men and 12 g/dL for women.^20^ Diabetes mellitus was defined as having at least 2 fasting blood sugar measurements of above 126 mg/dL or the use of antidiabetic agents.^21^ Hypertension was defined as a previous diagnosis of hypertension, a previous use of antihypertensive medications, or a systolic pressure exceeding 140 mm Hg and/or a diastolic pressure of over 90 mm Hg on at least 2 separate measurements during hospitalization.^22^

The SPSS software, version 22.0 (Armonk, NY: IBM Corp), was used for the statistical analyses. For the assessment of appropriate distribution characteristics and normality, mean (±standard deviation) was employed to express the continuous variables, and the *t*-test or the Mann–Whitney *U*-test was applied for group comparisons. The categorical variables were reported as numbers (percentages) and compared using the Fisher exact or the χ^2 ^test. Multivariate logistic regression analyses were performed to identify the independent predictors of CI-AKI using variables that showed marginal associations with CI-AKI in the univariate analyses. The receiver operating curve (ROC) was utilized to derive the cutoff value of the NLR for predicting CIN. A P value of less than 0.05 was considered statistically significant.

## Results

A total of 2000 patients with STEMI were retrospectively enrolled in the study. The mean age of the study population was 57.16±11.55 years. There were 1642 (82.1%) male and 358 female (17.9%) patients. The study population was divided into 2 groups: CI-AKI and non-CI-AKI. After primary PCI, 148 (7.4%) patients developed CI-AKI. The baseline demographic characteristics, laboratory findings, and angiographic and interventional data regarding the patients with and without CI-AKI are presented in Table 1. Being older, female, hypertensive, diabetic, hyperlipidemic, and anemic and having a prior cerebrovascular disease and LVEFs below 40% were associated with a higher likelihood of CI-AKI development (P<0.05 for all). A previous PCI procedure, aortocoronary bypass graft surgery, and the mean amount of the contrast medium used during the procedure were not statistically significantly different between the 2 groups. The patients with unsuccessful PCI procedures, chronic renal failure, and multivessel coronary disease were more likely to develop CI-AKI. Notably, the patients who developed CI-AKI after primary PCI had11-fold increase in-hospital mortality.

The mean NLR was significantly higher in the patients who developed CI-AKI after primary PCI than in those who did not (7.08±4.43 vs. 6.18±3.98; P=0.011). Age, the female gender, the baseline creatine level, hypertension, diabetes mellitus, chronic renal failure, and the NLR were the predictors of CI-AKI in the univariate analysis (Table 2). In the multivariate regression analysis, with the use of a model adjusted for the aforementioned parameters, age (odds ratio [OR]: 1.02, 95% confidence interval [CI]: 1.00–1.04, and P=0.034), hypertension (OR: 1.67, 95%CI: 1.14–2.46, and P=0.008), chronic renal failure (OR: 0.95, 95%CI: 0.93–0.97, and P=0.001), and an NLR of equal to or greater than 5 (OR: 1.78, 95%CI: 1.21–2.61, and P=0.003) were found to be independently correlated with the development of CI-AKI.

**Figure 1:**
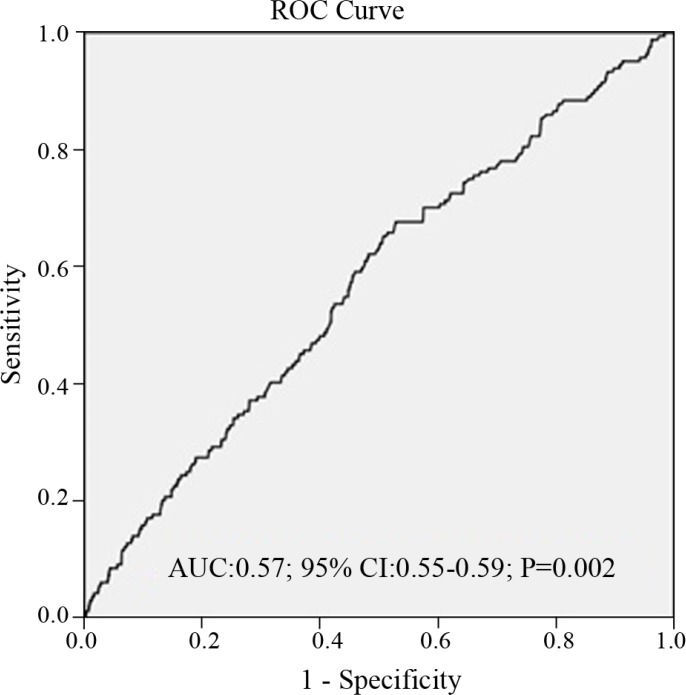
Receiver operating characteristic curve (ROC) analysis, revealing that the optimal cutoff value of the neutrophil-to-lymphocyte ratio (NLR) to predict contrast-induced acute kidney injury (CI-AKI) was ≥5 with 66% sensitivity and 47% specificity (area under curve: 0.57, 95%CI: 0.55–0.59, and P=0.002).

The ROC analysis showed that the best cutoff value of the NLR to predict CI-AKI was equal to or greater than 5, with 66% sensitivity and 47% specificity (area under the curve: 0.57, 95%CI: 0.55–0.59, and P=0.002) (Figure 1).

**Table 1:** Baseline demographic characteristics, laboratory findings, and angiographic and interventional outcomes regarding the patients with and without CI-AKI*

	**Non-CI-AKI Group** **(N=1852)**	**CI-AKI Group** **(N=148)**	**P**
**Age (y)**	56.48±11.20	65.73±12.40	**<0.001**
**Female gender**	318 (17.2)	40 (27.0)	**0.003**
**History **			
** Diabetes mellitus**	396 (21.4)	54 (36.5)	**<0.001**
**Hypertension**	528 (28.5)	76 (51.4)	**<0.001**
**Hyperlipidemia**	379 (20.4)	50 (33.7)	**<0.001**
** Prior PCI**	233 (12.6)	22 (14.9)	**0.501**
** Prior ACBG**	45 (2.4)	8 (5.4)	**0.054**
** Cerebrovascular accident**	16 (0.9)	9 (6.1)	**<0.001**
** Chronic renal failure**	132 (7.1)	49 (33.1)	**<0.001**
** Smoking**	706 (38.1)	38 (25.7)	**0.003**
** Anemia**	471 (25.4)	57 (38.5)	**<0.001**
**Laboratory and Echocardiographic Findings**			
** White blood cell count (cells/µL)**	11.96±3.65	12.41±4.61	**0.401**
** Hemoglobin (g/dL)**	13.63±1.65	12.94±2.11	**<0.001**
** Platelet (cells/µL)**	238.44±63.89	242.33±76.99	**0.986**
** Neutrophil (cells/µL)**	9.25±3.53	9.86±4.38	**0.150**
** Lymphocyte (cells/µL)**	1.89±0.95	1.74±0.98	**0.021**
** Monocyte (cells/µL)**	0.64±0.30	0.63±0.34	**0.375**
** Neutrophil-to-lymphocyte ratio (NLR)**	6.18±3.98	7.08±4.43	**0.011**
** Blood urea nitrogen (mg/dL)**	16.41±4.29	21.73±8.63	**<0.001**
** Baseline creatinine (mg/dL)**	0.83±0.22	1.18±0.39	**<0.001**
** Peak creatinine (mg/dL)**	0.94±0.31	1.88±0.97	**<0.001**
** eGFR (mL/min/1.73 m2)**	92.08±19.02	70.12±23.25	**<0.001**
** Ejection fraction <40%**	197 (15.8)	23 (24.5)	**0.042**
**Angiographic and Interventional Outcomes**			
** Total amount of contrast media used (mL)**	243.06±82.95	251.68±91.73	**0.327**
** Multivessel disease (>50%)**	352 (18.7)	39 (25.6)	**0.030**
** Stent implantation**	1570 (84.8)	112 (76.2)	**0.004**
** Stent length (mm)**	18.84±8.90	16.76±12.33	**0.235**
** Stent diameter (mm)**	2.55±1.24	2.45±1.28	**0.643**
** Successful PCI**	1483 (94.5)	96 (86.0)	**<0.001**
** Unsuccessful PCI**	87 (5.0)	16 (12.8)	**<0.001**
** In-hospital mortality**	18 (1.0)	16 (11)	**<0.001**

CI-AKI, Contrast-induced acute kidney injury; PCI, Percutaneous coronary intervention; ACBG, Aortocoronary bypass graft; eGFR, Estimated glomerular filtration rate

* Data are presented as mean±SD or n (%)

**Table 2:** Factors predicting CI-AKI in univariate and multivariate regression analyses

	Univariate Analysis			Multivariate Analysis		
	Odds Ratio	95% CI	P	Odds Ratio	95 % CI	P
Age	1.07	1.05-1.08	<0.001	1.02	1.00-1.04	0.034
Gender (female)	0.56	0.38-0.82	0.003	1.60	0.96-2.69	0.071
Basal creatinine	8.71	5.50-13.81	<0.001	0.70	0.25-1.94	0.501
Hypertension	2.64	1.88-3.71	<0.001	1.67	1.14-2.46	0.008
Diabetes mellitus	2.11	1.48-3.00	<0.001	1.17	0.78-1.75	0.448
CRF	0.95	0.94-0.96	<0.001	0.95	0.93-0.97	0.001
NLR ≥5	1.85	1.29-2.65	<0.001	1.78	1.21-2.61	0.003
Anemia	1.83	1.29-2.59	<0.001	0.93	0.62-1.39	0.744

All clinically relevant parameters were included in the model**. **

CI-AKI, Contrast-induced acute kidney injury; NLR, Neutrophil-to-lymphocyte ratio; CRF, Chronic renal failure

## Discussion

In the present study, we demonstrated that the NLR has an independent predictive value for the occurrence of CI-AKI in patients with STEMI treated via primary PCI. In particular, the patients whose minimum NLR was 5 had a 1.7-time higher risk for the development of CI-AKI. 

Coronary artery disease is the leading cause of death in developed countries.^23^ Inflammation and inflammation biomarkers, which have a key role in the pathogenesis of coronary artery disease and its adverse outcomes, have been investigated in many studies. Inflammation has also gained popularity in the pathogenesis of coronary artery ectasia and the coronary slow-flow phenomenon. Inflammation biomarkers such as white blood cells, acute-phase reactants, adhesion molecules, and cytokines have also been used to investigate the status of the inflammatory response in the body. C-reactive protein, an acute-phase protein, is the most investigated inflammation marker in this issue. It has been previously shown that increased levels of neutrophils are associated with the extent of myocardial injury and short-term prognoses in acute coronary syndrome.^24^ Additionally, lymphopenia, which is induced by acute stress during acute coronary syndrome, reflects the changes in the immune system. Moreover, lymphopenia, which is due to stress-induced cortisol release, is one of the earliest findings in acute coronary syndrome.^25^ An index that reflects both the acute state of the inflammation related to increased neutrophil levels and lymphopenia owing to acute physiological stress has been used in recent years. This index is obtained by dividing the absolute neutrophil count by the absolute lymphocyte count (NLR). The NLR is a simple and inexpensive index for assessing inflammation. The NLR, as a marker of inflammation, may play an important role in the pathogenesis of atherosclerosis.^26^ In addition, the NLR is an important predictor of mortality in patients with acute coronary syndrome, and both C-reactive protein and NLR levels have positive correlations with the no-reflow phenomenon in STEMI patients treated with primary PCI.^27^ Kaya et al.^28^ reported that a higher NLR in patients with STEMI treated via primary PCI was associated with increased mortality and major cardiac events.

CI-AKI is the third most common cause of acute renal failure in hospitalized patients.^6^^, ^^29^ The development of CI-AKI after STEMI is an independent predictor of long-term mortality and morbidity.^30^ Due to the increasing frequency of coronary angiography or PCI, the incidence of CI-AKI is on the rise. There are 2 main mechanisms responsible for the development of CI-AKI in patients with STEMI: medullary ischemia due to renal vasoconstriction and the direct cytotoxic effect of the contrast agent itself. After the injection of the contrast agent, there is an increased release of vasoconstrictor substances such as angiotensin, endothelin, and vasopressin; however, there is also a decreased release of vasodilator substances such as nitric oxide, all of which results in renal vasoconstriction and a decreased medullary blood flow.^3^^, ^^6^^, ^^7^ Some experimental studies have demonstrated a significant association between inflammation and the initiation and extension of CI-AKI.^31^ Ischemia, sepsis, or nephrotoxic agent exposure causes structural and functional changes in the renal vascular endothelium and tubular epithelial cells. Further, renal destruction is accentuated by the infiltration of macrophages, neutrophils, and natural killer cells. The hyperstimulation of neutrophils promotes the release of cytokines and the generation of reactive oxygen species, causing an increase in vascular permeability and endothelial dysfunction.^32^^, ^^33^ Activated neutrophils elevate the serum levels of arachidonic acid metabolites and, thus, reduce the response to vasodilatation and cause vasoconstriction, all of which lead to the stimulation of platelet adhesion and aggregation and result in the obstruction of renal capillary vessels. The upshot is a decrease in the blood flow to the renal tissue and the aggravation of the ischemic damage.^34^ Multiple risk factors have been shown to be related to CI-AKI, including diabetes mellitus and low hemoglobin levels**.**^19^ In the current study, even though the frequency of diabetes mellitus and the level of hemoglobin were different between the groups, these variables did not reach statistical significance in the multivariate analyses.

It has been known for some time that many different factors play a significant role in the development and progression of stable and unstable coronary artery disease and that the inflammatory process is one of the best-defined entities of all these factors.^35^^, ^^36^ White blood cells, including monocytes, lymphocytes, and neutrophils, play a critical role in the inflammatory response in the body. White blood cell counts and subtypes are defined as inflammatory markers in cardiovascular disease. The NLR has recently been shown to be a stronger inflammatory marker than the neutrophil count alone or the lymphocyte count alone.^37^ Zuin et al.^38^ demonstrated a significant correlation between the NLR and the SYNTAX score in patients with acute coronary syndrome treated via PCI. Previous research has also demonstrated that the NLR is an independent significant predictor of 1 year’s cardiovascular mortality in patients with acute coronary syndrome. Sawant et al.^39^ showed that the NLR based on an optimal cutoff value of 7.4 was an excellent predictor of short- and long-term survival in patients with STEMI. Akçay et al.^31^ reported that elevated C-reactive protein levels and high NLRs indicating an increased inflammatory response were associated with the onset and development of AKI. In a study conducted by Kurtul et al.,^40^ the results demonstrated that an NLR of equal to or greater than 3.46, the eGFR, and high sensitivity C-reactive protein were the independent predictors of the development of CI-AKI in patients with non-STEMI undergoing PCI. Elsewhere, Kaya et al.^41^ concluded that a higher NLR was the independent predictor of CI-AKI in 662 consecutive patients who underwent primary PCI with the diagnosis of STEMI. Our findings were concordant with those reported by Kaya and colleagues; nonetheless, our cohort was large by comparison with that in their investigation (662 patients vs. 2000 patients). Furthermore, the similar findings between the studies strengthen the notion that the NLR, as an inflammatory marker, plays a key role in predicting CI-AKI. Therefore, clinicians may use the NLR to assess the risk of CI-AKI development in patients with STEMI treated via primary PCI.

Our study has several limitations. We did not compare the NLR with other inflammatory markers such as fibrinogen, myeloperoxidase, and neutrophil gelatinase-associated lipocalin because they were not routinely measured in our study population. In addition, our investigation is a single-center, retrospective, and observational study with a limited number of patients and, as such, suffers from the inherent limitations of a retrospective design. Furthermore, we cannot rule out selection bias, although we spared no efforts to recruit consecutive patients. Moreover, due to the lack of data, we only evaluated the in-hospital all-cause mortality in the present study.

## Conclusion

The current study demonstrated that a higher NLR might have a predictive value for the occurrence of CI-AKI in patients with STEMI treated via primary PCI. The patients with a minimum NLR of 5 had a 1.78-time higher risk for the development of CI-AKI. Because our investigation is a retrospective single-center study, definitive suggestions could not be given based on only the NLR. However, the NLR may be combined with other markers such as neutrophil gelatinase-associated lipocalin for the prediction of CI-AKI in patients with STEMI undergoing primary PCI. In addition, in patients with STEMI and high admission NLRs candidated for primary PCI, it may be advisable that the initiation of early prophylactic measures for the prevention of CI-AKI be considered.

## Notes:

***This paper should be cited as: ***Tanık VO, Çınar T, Velibey Y, Öz A, Kalenderoğlu K, Gümüşdağ A, Aruğaslan E, Keskin M, Eren M. Neutrophil-to-Lymphocyte Ratio Predicts Contrast-Induced Acute Kidney Injury in Patients with ST-Elevation Myocardial Infarction Treated with Primary Percutaneous Coronary Intervention. J Teh Univ Heart Ctr 2019;14(2):59-66.
